# A novel olfactory pathway is essential for fast and efficient blood-feeding in mosquitoes

**DOI:** 10.1038/srep13444

**Published:** 2015-08-26

**Authors:** Je Won Jung, Seung-Jae Baeck, Haribalan Perumalsamy, Bill S. Hansson, Young-Joon Ahn, Hyung Wook Kwon

**Affiliations:** 1 WCU Biomodulation Major, Department of Agricultural Biotechnology, College of Agriculture & Life Sciences, and Research Institute of Agriculture and Life Sciences Seoul National University, Seoul, Republic of Korea; 2 Max Planck Institute for Chemical Ecology, Department of Evolutionary Neuroethology, Hans-Knoell-Strasse 8, D-07745 Jena, Germany

## Abstract

In mosquitoes, precise and efficient finding of a host animal is crucial for survival. One of the poorly understood aspects of mosquito blood-feeding behavior is how these insects target an optimal site in order to penetrate the skin and blood vessels without alerting the host animal. Here we provide new findings that a piercing structure of the mouthpart of the mosquitoes, the stylet, is an essential apparatus for the stage in blood feeding. Indeed, the stylet possesses a number of sensory hairs located at the tip of the stylet. These hairs house olfactory receptor neurons that express two conventional olfactory receptors of *Aedes aegypti* (AaOrs), AaOr8 and AaOr49, together with the odorant co-receptor (AaOrco). *In vivo* calcium imaging using transfected cell lines demonstrated that AaOr8 and AaOr49 were activated by volatile compounds present in blood. Inhibition of gene expression of these AaOrs delayed blood feeding behaviors of the mosquito. Taken together, we identified olfactory receptor neurons in the stylet involved in mosquito blood feeding behaviors, which in turn indicates that olfactory perception in the stylet is necessary and sufficient for mosquitoes to find host blood in order to rapidly acquire blood meals from a host animal.

Olfactory-driven behaviours enable mosquitoes to locate host animals from which they obtain blood meals essential to produce eggs. In the process, they transmit diseases to humans and animals^1^,^2^. Mosquito host-seeking behaviour can be dissected into sequential steps, each of which relies heavily on olfactory information from hosts^3^,^4^. Volatile compounds from animals such as 1-octen-3-ol and CO_2_ trigger flight orientation behaviour^5^. These volatiles are mainly detected by olfactory receptor neurons (ORNs) present in the maxillary palp of mosquitoes^6^ (Fig. S1). Less volatile host-emitted compounds, such as carboxylic acids, are critical to the mosquito’s ability to discriminate a suitable host from a non-preferred host animal at close range, e.g. in landing behaviour^7^,^8^. The final step of blood-feeding behaviour occurs when the stylet probes the host skin to obtain blood. This is a critical and dangerous moment for female mosquitoes, because unsuccessful probing might alert the host animal to their presence, which may result in considerable risks^9^. Therefore, mosquitoes try to locate blood vessels underneath the host skin rapidly and efficiently without provoking defense measures in the host (Fig. 1a). How is a quick and precise evaluation of the microtopography of blood vessels in the host skin possible? Here we hypothesized the existence of an additional chemosensory apparatus in the piercing-sucking stylet of the mosquito, which would help the insect locate blood vessels under the host skin (Fig. 1a). The stylet is a mouthpart appendage consisting of several structures^10^ (Fig. 1b), which penetrates the skin of host animals. The final stage of blood feeding is initiated by opening the host skin using the stylet mandibles and maxillae (Fig. 1b)^10^,^11^. Using molecular, behavioural, and neuroanatomical as well as electrophysiological approaches, here we show that two olfactory receptors, which are present in the stylet of the mosquito mouthpart, are sensitive detectors responding to volatile compounds in host blood and may play important role in final stage of host finding behaviors of mosquitoes. Also we demonstrate here that feeding behaviors of mosquitoes were affected by inhibiting gene expression of these stylet olfactory receptors. Our present analysis of novel olfactory function in the stylet of mosquito mouthpart thus provides a new insight of blood seeking behaviors and olfactory processing pathways in mosquitoes.

## Results

### Identification of additional chemosensory in the mosquito mouthpart

**Figure 1:**
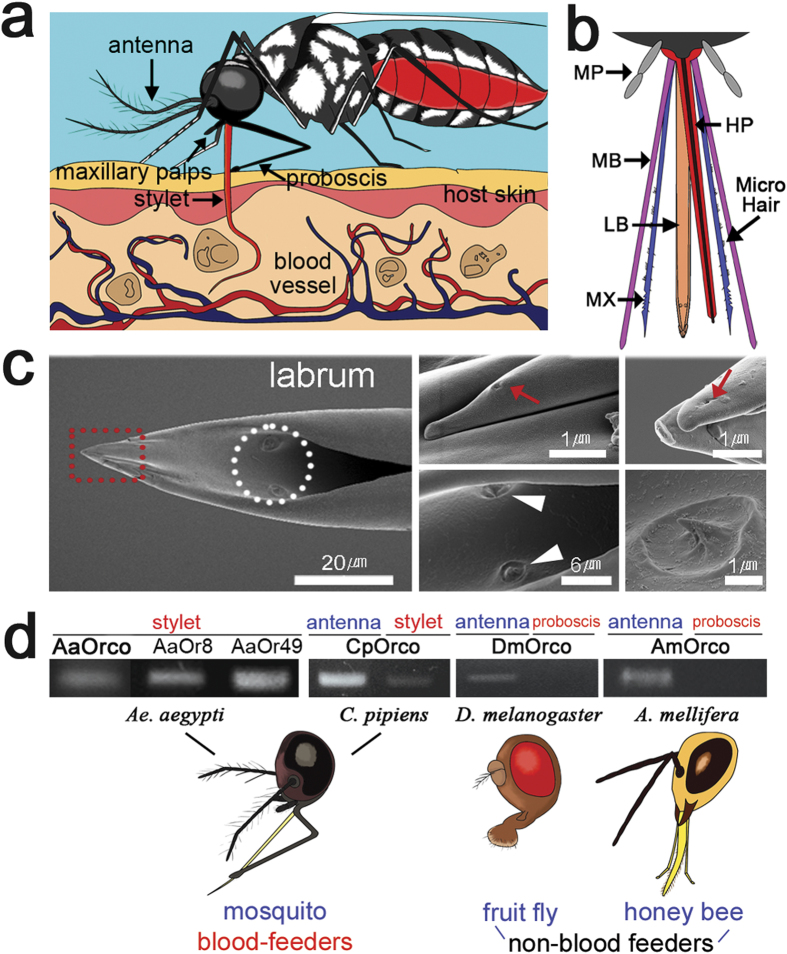
A novel olfactory organ in the mosquito stylet. (**a**) Schematic diagram of mosquito blood feeding. (**b**) The stylet consists of labrum (LB), hypopharynx (HP), and two pairs of mandibles (MBs) and maxilla (MX). (**c**) The tip area of LB. Pairs of the apical and subapical sensilla (dotted red box) with pores (arrows in second and third columns). Two campaniform sensilla (arrowheads in second column) of the LB (dotted circle). (**d**) The odorant receptor co-receptor (Orco) was identified in the mosquito stylet but not in non-blood feeding insects. Two odorant receptors, *AaOr8* and *AaOr49*, were identified in the mosquito stylet. This figure was created with a Flash Pro CS6 (Adobe) program by S. Baeck and H. Kwon.

As an evidence of potential olfactory reception in the stylet of the mosquito, we identified the presence of three pairs of sensory hairs, sensilla, at the tip of the labrum (LB) in the stylet of female *Aedes aegypti* mosquitoes (Fig. 1c). As identified by scanning electron microscopy, pores on the apical and subapical sensilla (Fig. 1c) indicated that these sensilla were likely to house chemosensory ORNs. A similar organization of sensilla in the stylet was also identified in mosquito species such as *Anopheles* and *Culex* mosquitoes (Fig. S2). By RT-PCR analysis, the olfactory receptor co-receptor (Orco)^12^ was shown to be expressed in the stylet of blood-sucking female mosquitoes. Orco acts as a dimerization counterpart to conventional, ligand-specific olfactory receptor (Or) proteins^13^ (Fig. 1d). In contrast, Orco was not detected in the proboscis (mouthparts) of other, non-blood feeding insects such as fruit flies (*Drosophila melanogaster*) and honey bees (*Apis mellifera*) (Fig. 1d), implying that blood-feeding mosquitoes exploit a unique olfactory pathway involving additional olfactory sensory organs that may play a pivotal role in securing blood meals from host animals. In order to probe for functional Or proteins and heat-related receptors in the stylet of *Aedes aegypti*, we screened sets of conventional Ors and heat-related receptors, such as TRPA1 and other TRP receptors (Fig. S3) previously reported to be expressed in the proboscis^14^,^15^. Among these Ors, *AaOr49* and *AaOr8* gene expression was detected in the stylet (Fig. 1d and Fig. S3B). *AaOr8* expression was not reported in the previous study^14^.

### Novel expression of odorant receptors in the stylet of the mosquito mouthpart

**Figure 2:**
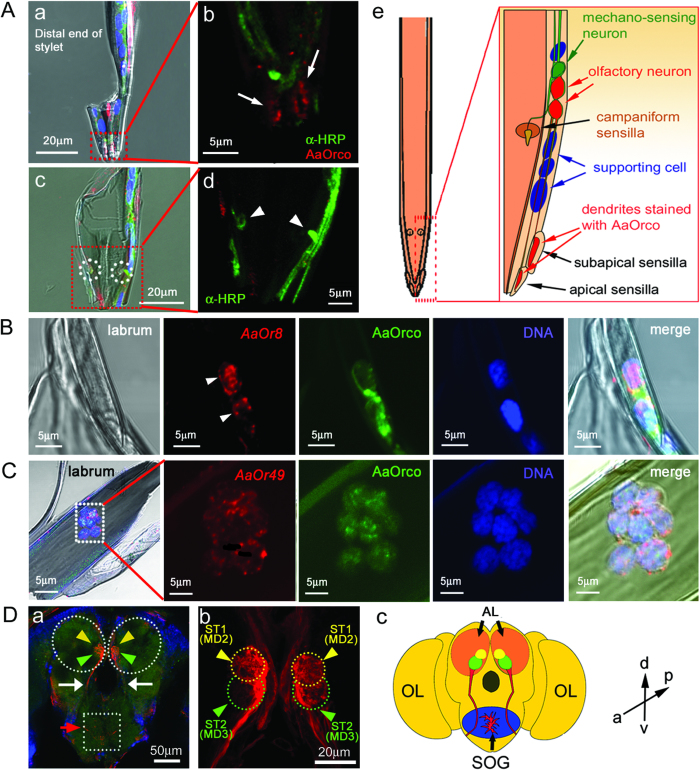
Stylet neurons are olfactory neurons. (**A**) Orco antibody (red) was localized to the distal part of the labrum of the stylet, where apical and subapical sensilla (red-dotted box and arrows in a, b) were innervated by Orco-expressing neurons, while the campaniform sensilla were not innervated by Orco neurons (white-dotted circles and arrowheads in c,d). b and d: magnified images of red-dotted boxes in a,c. Diagrams represent neuronal organizations in the labrum of the stylet in the mosquito mouthpart (e). (**B**) Localization of *AaOr8 gene* in the labrum using *in situ* hybridization. *AaOr8*-expressing neurons were localized (white arrowheads) in labrum and were co-localized with AaOrco (green). DNA staining with DAPI (blue) demonstrated that neurons were identified in the labrum of the mosquito stylet. (**C**) A cluster of *AaOr49* neurons was also identified in labrum (red), which were co-localized with AaOrco (green). (**D**) Neurons in the stylet of the mosquito sent their axons to two discrete glomeruli (arrowheads in a, b) of the antennal lobes (ALs) in the brain (white-dotted circles in a). Neuronal tracts from suboesophageal ganglion (SOG) to ALs were also observed (arrows in a). Projections from stylet to SOG were also observed (dotted box and red arrow in a). Pairs of MD2 (ST1) and MD3 (ST2) glomeruli (yellow and green dotted circles and arrows in b) in the ALs, targets of stylet olfactory receptor neurons (ORNs), were located in the ventro-medial part of the ALs by 3D reconstruction (MD2: yellow circles inside ALs; MD3: green circles inside ALs in c). Direction indicators in d represents as follows: a, anterior; p, posterior; v, ventral; d, dorsal. OL represents optic lobe.

In order to verify the expression of these stylet AaOrs on-site, we performed immunohistochemistry to localize the AaOrco receptor, using a *Drosophila* Orco (DmOrco) antibody. We found that apical and subapical sensilla located on the tip of the stylet labrum received dendritic arborizations from AaOrco neurons (red dotted box in Fig. 2A-a; arrows in Fig. 2A-b,A-e). In contrast, campaniform sensilla did not contain AaOrco-expressing neurons, implying that a pair of campaniform sensilla located on the inner side of stylet labrum in the mosquito would detect other sensory modalities such as mechanical or taste stimuli (Fig. 2A-c,d and Fig. S2). Campaniform sensilla may thus have other roles in feeding, e.g. tasting the quality of the blood, detecting positional information of blood vessels or sensing the flow of blood through the food canal^11^. Subsequently, we attempted to localize *AaOr8* and *AaOr49* genes in the stylet labrum using an *in situ* hybridization method, AaOr8 and AaOr49 expression was detected near the distal end of the stylet labrum, especially on the side of the labrum (Fig. 2B,C). In contrast, control experiments using sense RNA probes of these receptor genes did not show any notable staining patterns (Fig. S4).

ORNs of other appendages such as the antennae send their axons to the primary olfactory center of the brain, the antennal lobe (AL), homologous to the vertebrate olfactory bulb^16^. Having identified neurons of potential olfactory function in the stylet, we questioned whether these neurons expressing AaOr8 and AaOr49 convey sensory information to the AL. We employed an anterograde dye filling method as previously described^8^ and were able to show that the stylet ORNs indeed innervated the ALs. Two olfactory glomeruli in the ventro-medial part of the ALs received axonal branches from stylet ORNs. Axons of *AaOr8* and *AaOr49* expressing ORNs in the stylet terminate in specific glomeruli of the *Aedes aegypti* AL. Interestingly, compared with central projections to those of maxillary palp ORNs (Fig. S5), where *AaOr8* and *AaOr49* genes were also reported to be expressed^14^, we learned that two glomeruli, named MD2 and MD3^17^, are likely to share the axonal arborizations from stylet and maxillary palps (Fig. 2D and Fig. S5). It is conceivable that ORNs expressing AaOr8 and AaOr49 in the stylet labrum may have different functions in detecting chemosensory cues such as blood smell, while the stylet penetrates through the host skin and trace blood smell to locate a blood vessel precisely. It is likely that the ORNs in the maxillary palp help orient the host in long to mid range orientation, while the ORNs in the stylet precisely pinpoint skin penetration and blood vessel targetting. In addition to the central projection to ALs from stylet neurons, stylets also send neuronal information to the suboesophageal ganglion (SOG), known to process e.g. taste information and motor control of appendages in mouthparts^18^,^19^, indicating that mechanosensory and neurons may reside in the campaniform sensilla (Fig. 2D).

### Effects of blood-feeding behaviours by inhibition of AaOr8, AaOr49, and AaOrco gene expression

**Figure 3:**
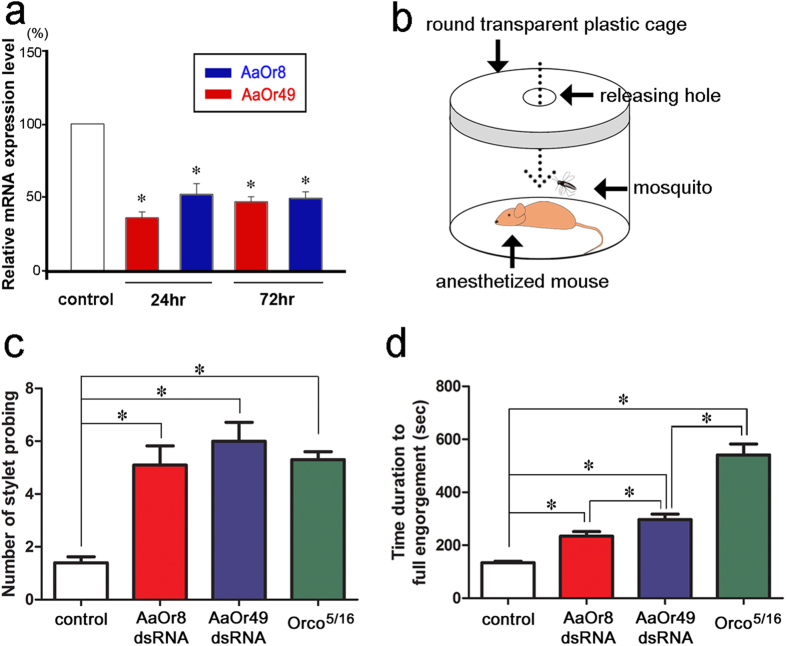
Effects of RNA interference of the *AaOr8* and *AaOr49* genes on blood-feeding behaviour in the mosquito. (**a**) Gene expression of *AaOr8* and *AaOr49* was strongly inhibited 24 and 72 h after dsRNA injections, compared to control, which received GFP (ANOVA, Tukey test, *p < 0.01, N = 10). (**b**) Schematic diagram of the blood-feeding bioassay. (**c**) Mosquitoes injected with dsRNAs of *AaOr8* and *AaOr49* as well as Orco mutant (Orco^5/16^) showed a significant delay in locating blood vessels compared to the control mosquitoes injected with GFP-dsRNA. (**d**) Mosquitoes injected with dsRNAs of *AaOr8* and *AaOr49* as well as Orco mutant (Orco^5/16^) represented the time delay to reach a full engorgement state for blood feeding, compared to the control mosquitoes injected with GFP-dsRNA (ANOVA, Tukey test, *p < 0.05, N = 10). Mouse and mosquito in Fig. 3b were created with a Flash Pro CS6 (Adobe) program by S. Baeck and H. Kwon.

In order to elucidate the olfactory functions, we proceeded to examine whether RNA interference of these stylet AaOr gene expression would affect blood-feeding behaviour in the mosquitoes. First, we found that RNA expression of *AaOr8* and *AaOr49* genes in the stylet was down-regulated with about 50% even at a 72 h time point after dsRNA injections, compared to GFP-dsRNA-injected mosquitoes (Fig. 3a). In a behavioural assay (Fig. 3b), we measured the number of stylet probings into the skin of a live mouse and the time duration from first insertion of stylet into the mouse skin to full engorgement. (Fig. 3c,d). Intriguingly, *AaOr8*- and *AaOr49*-dsRNA-injected mosquitoes and *AaOrco* mutant mosquitoes (Orco^5/16^)^20^ demonstrated highly repeated stylet probing (Fig. 3c and Videos S2 ~ S4), compared to GFP-dsRNA-injected control mosquitoes (Fig. 3c and Video S1). Moreover, the time required to full engorgement was significantly extended in *AaOr8*- and *AaOr49*-dsRNA-injected mosquitoes as well as *AaOrco* mutant mosquitoes (Orco^5/16^) (Fig. 3d and Videos S1 ~ S4). Both these facts support the idea that -AaOr8 and AaOr49 are important for blood feeding behaviours. The exact roles of the narrowly tuned AaOr8 and the more broadly tuned AaOr49 in blood feeding remain to be investigated.

### Functional study of AaOr8 and AaOr49

**Figure 4:**
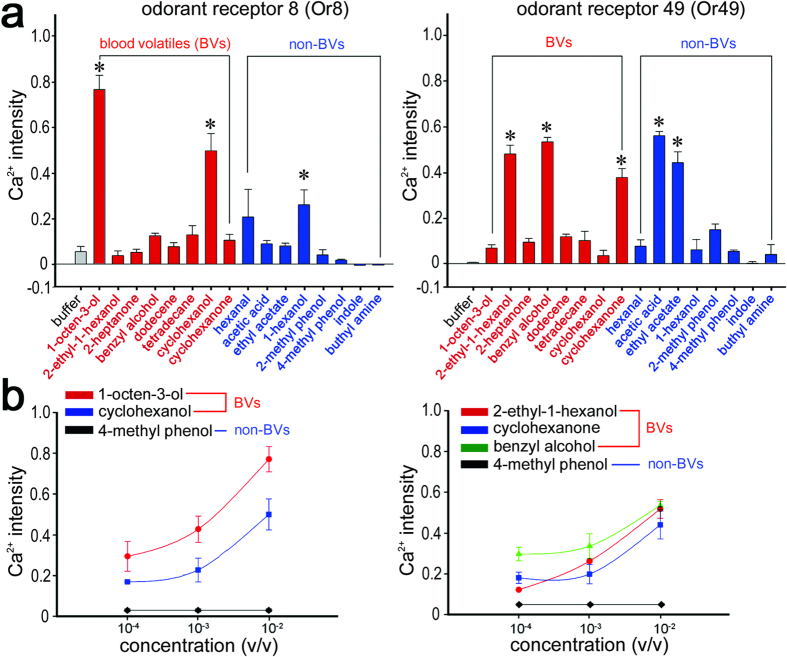
Functional study of AaOr8 and AaOr49 as receptors for blood volatiles (BVs). (**a**) *In vitro* calcium imaging using Sf9 cells expressing AaOr8 and AaOr49 together with AaOrco showed calcium influx responses to several BV and non-BV compounds. Sf9 cells expressing AaOr8/AaOrco was strong responses to BV compounds such as 1-octen-3-ol and cyclohexanol and showed weak responses to non-BVs such as 1-hexanol (p < 0.05, N = 4), while Sf9 cells expressing AaOr49/AaOrco responded to a broad spectrum of both BVs and non-BVs such as 2-ethyl-1-hexanol, benzyl alcohol, cyclohexanone, acetic acid, and ethyl acetate (p < 0.05, N = 4). (**b**) Dose-dependent calcium influx activity after stimulation with active BV ligands for AaOr8- and AaOr49-expressing Sf9 cells. 4-methyl phenol showed no calcium influx to both olfactory receptors.

Subsequently, we expressed AaOr8 and AaOr49 proteins in a heterologous Sf9 expression system for live calcium imaging to reveal the olfactory function of AaOr8 and AaOr49^21^. Several volatile compounds of human blood, including 1-octen-3-ol and other blood-signature volatiles, have been shown to be detected by mosquitoes^22^. Here we identified a number of additional volatile compounds from horse blood (blood volatiles [BV]), such as cyclohexanol, cyclohexanone, and 2-ethyl-1-hexanol (Fig. S6A). Therefore, we tested ligand specificity of AaOr8 and AaOr49 to 16 chemical candidates (8 BV and 8 non-BV compounds) using a heterologous expression system of Sf9 cell lines. Obviously, AaOr8 and AaOr49 expressed in the heterologous expression system clearly showed olfactory responses elicited by both BVs and non-BV odors (Fig. 4; Figs. S6 and S7). AaOR8 has been already known as to binding with 1-octen-3-ol^23^. Indeed, AaOr8 was narrowly tuned to BVs such as 1-octen-3-ol and cyclohexanol as well as weak responses to 1-hexanol in our study (Fig. 4a and Videos S5 ~ S6), while AaOr49 represented rather broadly tuned olfactory profiles to several BV (2-ethyl-1-hexanol, benzyl alcohol, and cyclohexanone) and non-BV (acetic acid and ethyl acetate) compounds (Fig. 4a; Fig. S6C,D and Videos S7 ~ S8). Contrary to the non-responsive control lines (Fig. S6B), the Sf9 cells expressing the two Ors responded to all activating ligands in a dose-dependent manner (Fig. 4b). Previous functional studies of AaOr8^24^ and *Anopheles gambiae* Or8 (AgOr8)^25^ demonstrated that Or8 was extremely narrowly tuned to 1-octen-3-ol and even discriminated among enantiomers of this compound; functional studies on AaOr49, on the other hand, have not been reported. Therefore, AaOr8 and AaOr49 present in the stylet would have discrete functions to locate a blood source after the mosquito mouthparts penetrate the host skin (Fig. 1a and Fig. S1).

## Discussion

In nature, animal mouthparts show diverse adaptations to ecological niches and diets^26^,^27^,^28^. Host shifts have provided several scenarios where the olfactory apparatus connected to mouthparts of blood-feeding mosquitoes has adapted to host animals^2^,^10^. One mechanism involved in host shifts has been proposed to be the loss of Or genes for detecting repellent chemicals^29^,^30^. Here we reveal a novel olfactory pathway, which in evolutionary history could be an important factor driving female mosquitoes to shift the hosts from which they obtain blood meals. Evolutionarily, the Or genes expressed in the stylet, AaOr8 and AaOr49, seem to be more derived than those of orthologous Or genes in insects that consume plants and nectar such as aphids and honey bees (Fig. S8). It is thus likely that these Or genes may be key evolutionary determinants in the shift from feeding on plant juice to hematophagy^31^. It will be interesting to investigate whether plant-sap feeders such as male mosquitoes and aphids also have a similar olfactory pathway via the proboscis.

Taken together, the ability to precisely and efficiently probe blood vessels under the host skin should be under strong positive selection in blood-feeding insects with piercing mouthparts. Olfactory functions must interact with taste and mechanical sensation, and optimal saliva composition in the stylet to ensure optimal feeding^1^,^32^. These interactions remain to be investigated and will in turn lead to an even better understanding of how precisely mosquitoes guide their bite on a warm-blooded animal and how this ability has evolved. Based on our present understanding of blood sensing in mosquitoes, it would be further feasible that a genetic disruption of stylet-associated sensory pathways employing targeted manipulation of receptors^31^ could have the potential to reduce mosquito biting and consequently be of importance for human health.

## Methods

### Mosquito preparation

An insecticide-susceptible strain of *Aedes aegypti* was reared in the laboratory. Larvae were reared in plastic trays (25 × 35 × 5 cm), and provided with distilled water and 0.5 g of sterilized diet (40-mesh chick chow powder and yeast, 4:1, w/w). Pupae were collected and transferred into plastic cups containing distilled water and placed in transparent plastic cages (25 × 25 × 25 cm) with polyester netting in the front panel. Mosquitoes were reared at 27 ± 1 °C with 70 ± 5% relative humidity under a 12:12 h (L:D) photoperiod. Non-blood-fed 5- to 7-day-old female mosquitoes were used in the experiments.

### RNA extraction, cDNA synthesis, RT-PCR

Total RNAs were extracted from the stylets and heads of female mosquitoes using a Qiagen RNeasy kit according to the manufacturer’s instructions (Qiagen, Valencia, CA, USA), after which RQ1 RNase-free DNase I was applied according to the manufacturer’s instructions (Promega, Madison, WI, USA). Reverse transcription procedures were carried out as described previously^8^.

### *In situ* hybridization and immunostaining

RNA probes of *AaOR8* and *AaOR49* for *in situ* hybridization were prepared using DNA clones obtained from RT-PCR as mentioned above. Digoxigenin-labeled RNA probes were generated for sense and antisense using SP6 and T7 RNA polymerases, depending on the direction of an inserted PCR product to pGEM-T-easy vector (Promega, Madison, WI, USA). Detailed procedures for probe preparation and procedure for *in situ* hybridization were followed as described previously^8^. Prepared mosquito stylet samples were directly immersed in 4% PFA and incubated at 4 °C. Paraffin-embedded preparations of adult mosquito stylets were sectioned at 4 ~ 5 μm thickness by using a microtome (HM340E, Microm). Sections were dried at 40 °C overnight and subsequently dewaxed with Citri-Solv (Fisher BioSciences, USA) and rehydrated with a series of ethanol to PBS buffer as described previously^30^. Signals were visualized by peroxidase (POD) coupled to anti-DIG antibodies (Roche, Indianapolis, IN) at 1:200 dilution. POD signals were detected by using a tyramid signal amplification (TSA) kit according to the manufacturer’s instructions (PerkinElmer, Waltham, MA, USA). Subsequently, immunostaining using DmOrco antibody and AlexaFluor 488 palloidin (Invitrogen) were carried out with a blocking solution consisting of 3% normal goat serum in PBS solution. DmOrco antibody was diluted 1:2000 in PBS with 0.1% Tween 20 and incubated at 4 °C overnight. Samples were washed with PBS solution three times and mounted with mounting medium (Vectashield with DAPI, H-1200, Vector Laboratories, Burlingame, CA, USA). Fluorescent images were captured using a LSM 700 confocal microscope (Zeiss, Germany).

### Anterograde dye filling

Anterograde filling using neurobiotin was performed as described previously^8^. The tip area of the stylet was severed without any damage to the shaft and labium of the proboscis, and the remaining stylet part was immediately immersed in a glass electrode filled with 1% neurobiotin (Vector Laboratories, Burlingame, CA, USA). Mosquitoes were kept humid at 4 °C for 10 ~ 12 h, after which they were decapitated and heads were fixed in 4% paraformaldehyde (PFA) solution at 4 °C overnight. Brains were dissected and washed in PBS for 2 ~ 4 h in the dark, followed by dehydration with a 0 ~ 100% ethanol series to 100% propylene oxide (Sigma, St. Louis, USA) for 5 min. Brains were then rehydrated to PBS and incubated with a 1:50 dilution of streptavidin–Alexa Fluor 594 conjugate (Molecular Probes, CA, USA) at 4 °C overnight. Whole mounts of the brain were washed in PBS for 1 ~ 2 h and mounted on a glass slide with Vectashield with DAPI (H-1200, Vector Laboratories). For plastic section in Spurr’s resin, further procedures were followed as described previously^33^. Whole mounts and resin sections of mosquito brains were observed using LSM 700 confocal scanning microscope (Zeiss, Oberkochen, Germany)

### Scanning electron microscopy (SEM)

Mosquito heads with stylet were prepared by removing only the proboscis part (labium and shaft). Samples were washed by using PBST 0.1% and were dehydrated with a series of alcohol to 100%, after which each head was desiccated in a 40 °C dry oven. The basal side of the head was attached to a strut-coated double sided adhesive tape (Agar Scientific, Essex, United Kingdom). Resolution of a scanning electron microscopy (SUPRA 55UP, Zeiss, Oberkochen, Germany) was about 1.0 nm (15 kV) to 1.7 nm (1 kV).

### Identification of blood-originated volatiles

A Clarus 680 gas chromatograph (PerkinElmer, Fort Belvoir, VA) was used to separate and detect the volatile constituents of horse blood (Innovative Research Inc. Novi, MI, USA). In order to extract blood volatiles, horse blood (1 ml) and hexane (1 ml) were mixed after which volatile compounds were collected from supernatants. Analytes were separated with a Perkin-Elemer 60 m × 0.25 mm ID (*d*_f_ = 0.25 μm) DB-5MS capillary column (Folsom, CA). The flow velocity of the helium carrier gas was 1.0 mL min^−1^. The oven temperature was kept at 50 °C (5 min isothermal) and programmed to 280 °C at a rate of 2 °C min^−1^, then isothermal at 280 °C for 10 min. The injector temperature was 280 °C. The linear velocity of the helium carrier gas was 24.4 cm s^−1^ (30 °C) at a split ratio of 1:50. Chemical constituents were identified by co-elution of synthetic samples following co-injection. Gas chromatography-mass spectrometry (GC-MS) analysis was performed using a Clarus 680T gas chromatograph-mass spectrometer (PerkinElmer, Fort Belvoir, VA). The capillary column and temperature conditions for the GC-MS analysis were the same as described above for the GC analysis. The ion source temperature was 250 °C. The interface temperature was kept at 260 °C, and mass spectra were obtained at 70 eV. The sector mass analyser was set to scan from 35 to 550 amu every 0.2 s. Chemical constituents were identified by comparison of mass spectra of each peak with those of authentic samples in a mass spectrum NIST 11 library (sisweb.com/nist).

### Heterologous expression of AaOrco, AaOr8, and AaOr49 into Sf9 cells

Sf9 cells were cultured in an incubator (Thermo scientific, OH, USA) at a constant temperature of 24 to 25 °C. SF900-II media (Invitrogen, CA, USA) was used to culture cells. Sf9 cells were transfected with full coding sequences of cDNA of odorant receptors (AaOrco, AaOr8, and AaOr49) of *Ae. aegypti*, which were inserted into pIB/V5-His vector using Cellfectin® II Reagent (Invitrogen, Grand Island, NY, USA). Transfected cell lines were cultured in 10 μg/ml blastcidine Sf900-II media (Invitrogen, Grand Island, NY, USA). Full length cDNAs were synthesized from mRNA extracted from the stylet with Superscript III (Invitrogen, CA, USA) using gene specific primers (Table S1). The expression vector plasmids were synthesized by inserting cDNAs of AaOrco, AaOr8, and AaOr49 into multiple cloning sites of a pIB/V5-His vector (Invitrogen, CA, USA) using the restriction enzymes NotI and XbaI (Koscamco, Anyang, Korea). Template pDNA for pIB-Or8 and Or49 and primer were then mixed with kit solutions and the total PCR reaction volume and conditions were followed as described earlier^34^.

### Calcium imaging

Transfected cell lines were subsequently cultured on confocal dishes (SPL, Pocheon, Korea). Before experiments, the culture media was removed and 100 μl of 2 μM Fluo-4 AM were loaded and directly replaced (Invitrogen, Grand Island, NY, USA), after which the cells were incubated in the dark for 90 min at room temperature. After incubation, 1X HBSS buffer was added onto the confocal dish, which was then directly placed in the LSM700 inverted confocal microscope for observation (Zeiss, Oberkochen, Germany). Images were captured maximum 100 frames per every two second interval. Test odorant chemicals were dissolved in dimethylsulfoxide (DMSO) at 10^−4^ ~ 10^−2^ M concentrations. Calcium influx into the transfected cells upon ligand binding was monitored and analyzed with ZEN software (Zeiss, Oberkochen, Germany). Fluorescent intensity changes were calculated as follows^34^: Intensity F = (F_after_ − F_before_)/F max.

### dsRNA synthesis

Partial sequences of *AaOr8* and *AaOr49* were sub-cloned from full coding cDNA using specific primers (Table S1) in pGEM-T Easy vector (Promega, Madison, WI, USA). Using *AaOr8*- and *AaOr49*- gene specific primers with T7 promoter sequence with Megascript RNAi kit (Invitrogen, Carlsbad, CA, USA), PCR products of each gene was amplified directly (Table S1), after which the PCR product was gel-extracted using QIAquick Gel Extraction kit (Qiagen, Valencia, CA, USA). GFP gene was also subcloned into pGEM-T vector and its dsRNA was directly amplified with the same procedure. dsRNAs of *AaOr8, AaOr49,* and *GFP* were then synthesized using Megascript RNAi kit according to the manufacturer’s instructions (Invitrogen, Grand Island, NY, USA).

### Injection of dsRNA for target gene silencing

1 ~ 2-day-old cold-anesthetized mosquitoes were injected into the thorax with dsRNAs by using a nano-injector (Nanoliter 2000, WPI, Sarasota, Florida, USA). 1 μg of dsRNAs was injected for better silencing efficiency of the *AaOr8* and *AaOr49* target genes^35^. The same amount of GFP dsRNAs was used for control dsRNA injection. The gene silencing effect was verified using quantitative real-time PCR (qRT-PCR). Total RNAs were extracted from injected mosquitoes as described above. 1 μg of RNA was used for cDNA synthesis, using the superscript^tm^ III (Invitrogen, Grand Island, NY, USA). qRT-PCR was conducted with the StepOnePlus (Applied Biosystems, CA, USA) using SYBR green qPCR Master Mix (Fermentas, Ontario, Canada) with gene-specific primers (Table S1). Quantitative analysis of the gene silencing capacity was assessed by employing a StepOne plus Software V2.0 (Applied Biosystems). Analyzed results were normalized to the validated control gene, *rps7* gene of *Ae. aegypti*, using the ΔΔCt method^36^.

### Behavioural assay

Blood-feeding bioassays were conducted with AaOr8-, AaOR49-dsRNA injected mosquitoes and control mosquitoes (dsRNA-GFP-injected and Orco mutant mosquitoes). dsRNAs were injected into mosquitoes with a Nanoliter 2000 injector (WPI, Sarasota, FL, USA). Five days after dsRNA injection, the blood-feeding behaviour of female mosquitoes was investigated using a seven week old SKH1-hairless mouse, which was anesthetized by intraperitoneal (IP) injection with a mixture solution of Zoletil (Virbac, Texas, USA) and Rompun (Bayer, Seoul, Korea) (20 μl of Zoletil at 100 mg/ml and 60 μl of Rompun at 23.3 mg/ml in PBS) using 1 ml Kovax disposable syringes (Korea Vaccine Co. Ansan, Korea). After complete anesthetization, the mouse was placed on the bottom of a transparent plastic jar (ID = 16 cm) (Fig. 4b). With blood-feeding behavioural assays, the number of stylet probing and time duration to finish flood feeding (time to full engorgement in a tested mosquito) were measured. Animal care and handling procedures in this experiment were approved by the Institutional Animal Care and Use Committees (IACUC) of Seoul National University, Korea (Approval number: SNU-121214-1). All experiments were performed in accordance with relevant guidelines and regulations.

## Additional Information

**How to cite this article**: Jung, J. *et al.* A novel olfactory pathway is essential for fast and efficient blood-feeding in mosquitoes. *Sci. Rep.* **5**, 13444; doi: 10.1038/srep13444 (2015).

## Supplementary Material

Supplementary Information

Supplementary Video 1

Supplementary Video 2

Supplementary Video 3

Supplementary Video 4

Supplementary Video 5

Supplementary Video 6

Supplementary Video 7

Supplementary Video 8
