# Supplementary material for: A novel olfactory pathway is essential for fast and efficient blood-feeding in mosquitoes
Source: Sci Rep. 2015 Aug 26;5:13444. doi: 10.1038/srep13444 (PMC4549640; doi:10.1038/srep13444)
Supplement: Supplementary Information [file srep13444-s1.pdf]

Supplemental Information

A novel olfactory pathway is essential for fast and efficient blood-feeding in mosquitoes

Je Won Jung[1], Seung-Jae Baeck[1], Haribalan Perumalsamy[1], Bill S. Hansson[2], Young-Joon Ahn[1], Hyung Wook Kwon[1]*

[1] WCU Biomodulation Major, Department of Agricultural Biotechnology, College of Agriculture & Life Sciences, Seoul National University, Seoul, Republic of Korea

[2] Max Planck Institute for Chemical Ecology, Department of Evolutionary Neuroethology, Hans-Knoell-Strasse 8, D-07745 Jena, Germany

*Correspondence to: biomodeling@snu.ac.kr

Phone: +82-2-880-4915

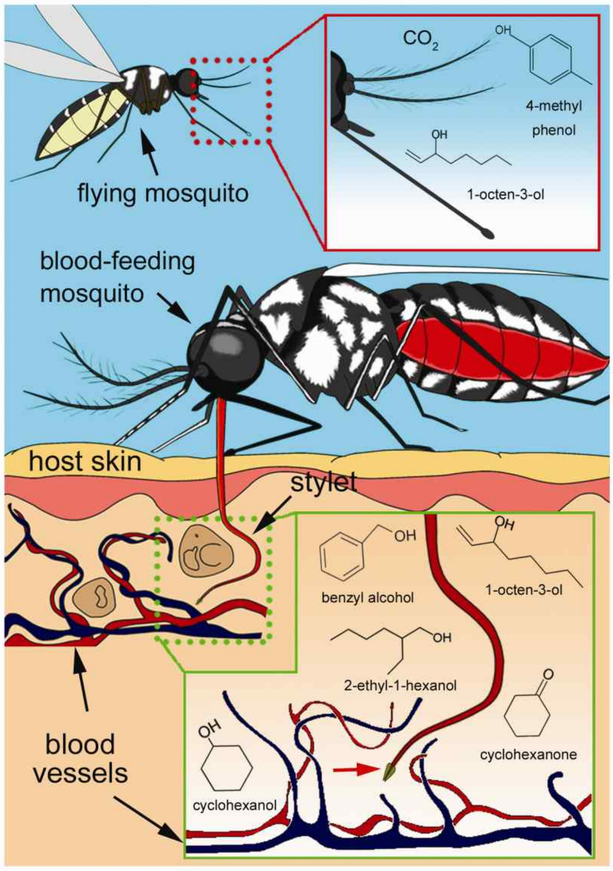

Figure S1. Key olfactory cues from a host animal during host finding behavior in mosquitoes. Mosquitoes detect volatile compounds in order to locate host animals for blood feeding. At a distance, a flying female mosquito detects highly volatile chemicals from the host such as 4-methyl phenol, 2-methyl phenol, CO_2 and 1-octen-3-ol through antenna and maxillary palps (top panel). In contrast, a landed mosquito on host skin should locate an optimal feeding site and blood vessels to take blood very precisely. Here we show that the mosquito uses an additional chemosensory organ to detect blood-related volatiles such as benzyl alcohol, 2-ethyl-1-hexanol, cyclohexanone, cyclohexanol, and 1-octen-3-ol. A red arrow indicates olfactory sensilla on the distal end of the stylet, which may play a crucial role in locating blood vessels of a host animal precisely. This figure was created with a Flash Pro CS6 (Adobe) program by S. Baeck and H. Kwon.

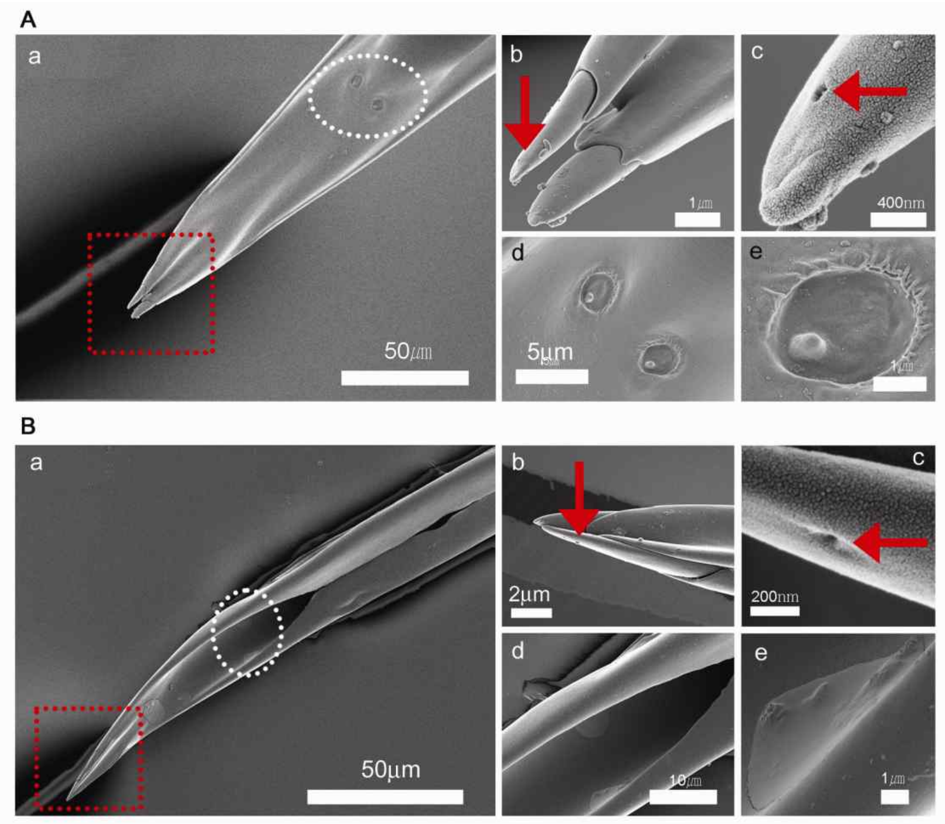

Figure S2. Scanning electron micrographs of the labrum in two other mosquito species. (**A**) The labrum of the stylet in *Anopheles sinensis*, a malaria vector mosquito*,* also possesses apical sensilla (red dotted box in a). Pores on the lateral area of the sensilla were also identified (arrows in b, c), indicating that these sensilla detect odor information. Similarly with *Aedes aegypti*, campaniform sensilla (d, e) were also identified on the inner surface of the labrum (circle in a). (**B**) *Culex pipiens pallens* mosquito show the same patterns at the tip of the labrum (a-e).

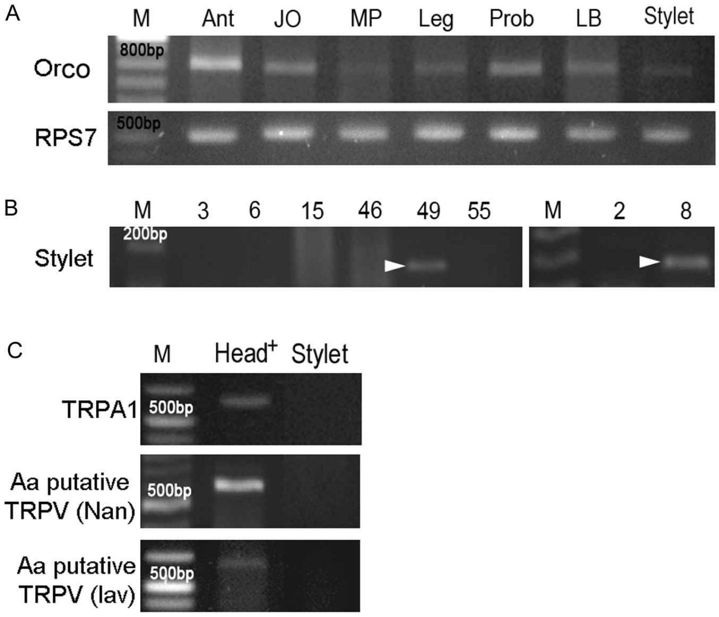

Figure S3. Expression patterns of odorant receptors in the stylet of *Aedes aegypti* (**A**) Expression patterns of the *Ae. aegypti* odorant receptor co-receptor (AaOrco) gene in the sensory organs. M: 100bp DNA ladder, Ant: antenna; JO: Johnston's organ; Prob: proboscis, LB: labium of the proboscis. (**B**) The expression patterns of odorant receptors (Ors) in the stylet of *Ae. aegypti*. According to previously reported AaOrs in the proboscis, which may include the stylet, we identified *AaOr8* and *AaOr49* in the stylet (arrowheads). (**C**) Expression patterns of transient receptor potential (TRP) channels in the stylet. No TRP receptors such as TRPA1 and two putative TRPV genes expression orthologous to *Drosophila* TRPV1 genes, *nanchung* and *inactive* were expressed in the stylet. Control parts depicted as Head$^+$ which include head and sensory appendage except stylet showed TRP expression.

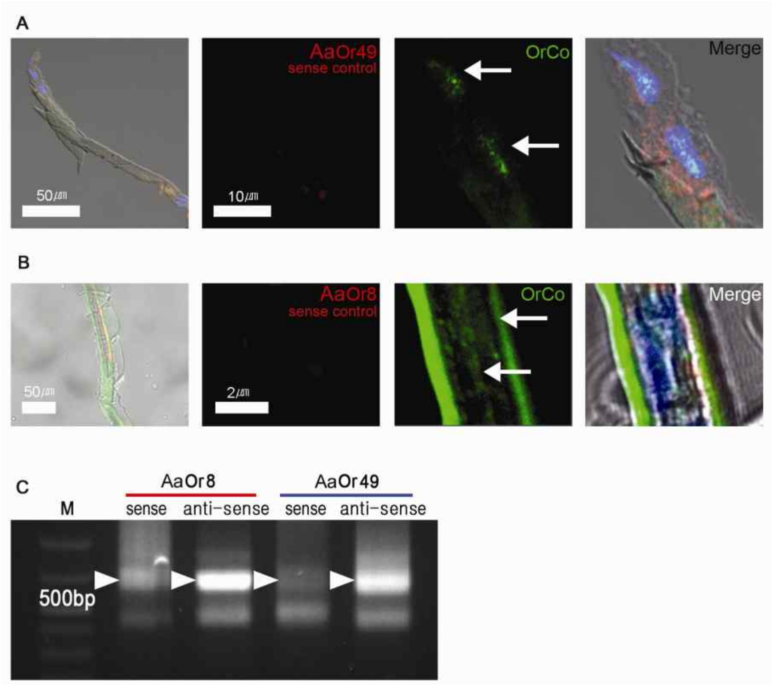

Figure S4. Sense RNA probe control of in situ hybridization of *AaOr8* and *AaOr49* and AaOrco antibody staining in the stylet of *Ae. aegypti.* (**A**) Control *in situ* hybridization with sense RNA probes of *AaOr49* (red), where AaOrco-positive neurons were expressed in the labrum of the stylet. (**B**) Control *in situ* hybridization with sense RNA probes of *AaOr49* (red). AaOrco-expressing neurons were localized in the labrum of the stylet. (**C**) Preparation of RNA probes were verified in the running gel before application to in situ hybridization. The size of *AaOr8* and *AaOr49* was 568bp and 560bp, respectively.

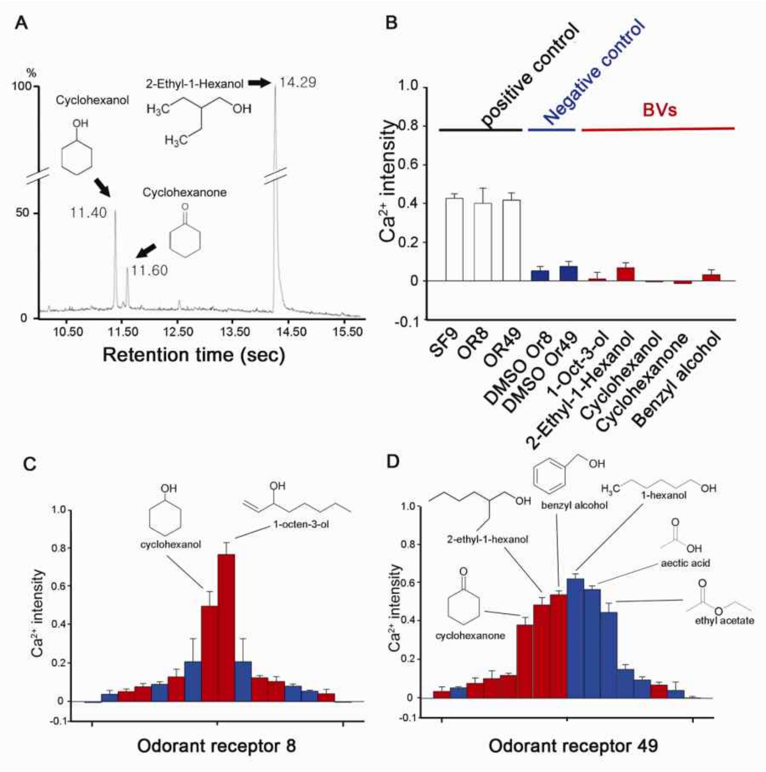

Figure S5. Characterization of volatile compounds from horse blood and activities to cell lines transfected with *AaOr8* and *AaOr49*. (**A**) Major blood volatiles (BVs) from horse blood analyzed by GC/MS analysis. Three prominent volatile compounds were confirmed. These were cyclohexanol, cyclohexanone, and 2-ethyl-1-hexanol. (**B**) Positive and negative controls of calcium imaging analysis using non-transfected and *AaOr8*- and *AaOr49*- transfected Sf9 cells to positive compounds (calcium ionophore) and to various volatile compounds as well as solvents such as DMSO. The calcium ionophore activated both transfected and non-transfected cell lines (white; positive control). BVs did not elicit calcium influx in non-transfected Sf9 cell lines (n=3~4). (**C, D**) Summary histogram representing olfactory tuning breadth of AaOr8 and AaOr49 to BVs and non-BVs chemicals. AaOr8 showed narrow tuning focused on 1-octen-3-ol and cyclohexanol, while AaOr49 had a rather broad tuning to BV (red) and non-BV (blue) compounds.

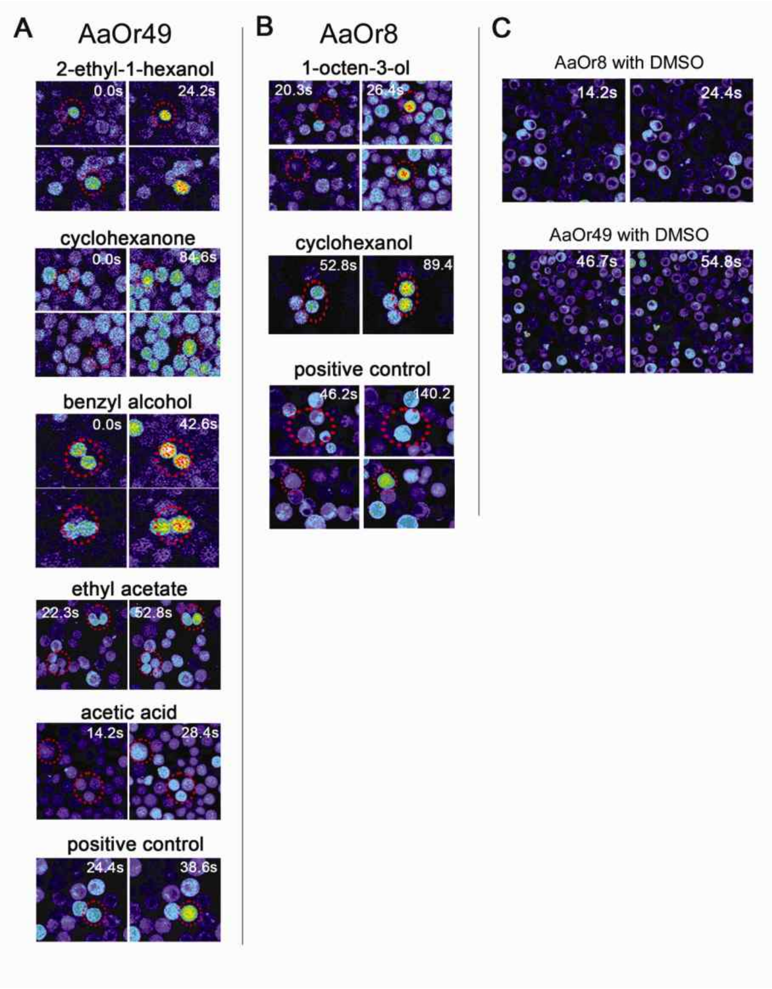

Figure S6. Representative real-time calcium images of cellular responses to several blood-associated volatile compounds and positive and negative control (DMSO) of the in Sf9 cells expressing AaOr8 or AaOr49, co-transfected with AaOrco. (**A**) Responses of calcium influx of AaOr49-tranfected cell lines to 2-ethyl-1-hexanol, cyclohexanone, benzyl alcohol, ethyl acetate, and acetic acid as well as positive control. (**B**) Responses of calcium influx of AaOr8-tranfected cell lines to 1-octen-3-ol, cyclohexanol, and positive control. (**C**) Responses of calcium influx of AaOr8- and AaOr49-tranfected cell lines to the negative control (DMSO). Numbers inside each figure represent real time point after chemical stimulation. Red dotted circles indicate most strongly responsive cells.

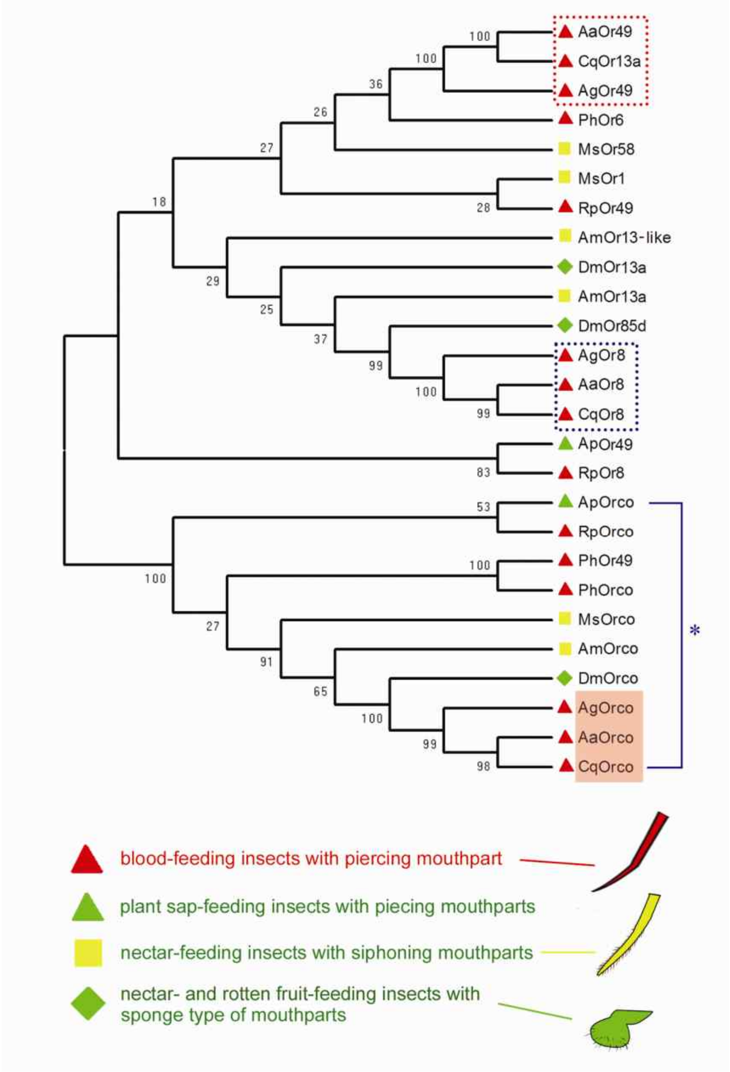

Figure S7. Phylogenetic relationships of orthologous genes of AaOrco, AaOr8, and AaOr49 in several insect species with specialized mouthparts. The phylogenetic tree was reconstructed based on homologous genes to AaOr8 and AaOr49. * indicates the highly conserved Orco cluster. The mosquito Orco is depicted as a red-highlighted box. Red triangles indicate insects with an elongated and piercing type of mouthparts for blood-feeding. Green triangles indicate insects with piercing type of mouthpart for plant-sap feeding such as in aphids. Yellow squares represent siphoning and nectar-feeding insects such as honey bees (*Apis mellifera*) and moths (*Manduca sexta*). Green diamonds indicate insects with a sponging type of

mouthparts such as fruit flies. Orthologous genes in other mosquitoes to AaOr8 and AaOr49 were divided into two clusters (blue and red dotted boxes, respectively), indicating that these receptors may be relevant for specific behaviors such as blood-feeding. Ap, *Acythosiphon pisum*; Aa, *Ae. aegypti*; Ag, *Anopheles gambiae*; Am, *Apis mellifera*; Cq, *Culex quinquefasciatus*; Dm, *Drosophila melanogaster*; Ms, *Manduca sexta*; Ph, *Pediculus humanus corporis*; Rp, *Rhodnius prolixus.*

Table S1. List of gene-specific primers used for RT-PCR, full-coding sequencing, dsRNA construction, and preparation for RNA probes for *in situ* hybridization. Gene accession numbers were retrieved from the genome database for each given species as noted below. Partial sequences of the odorant receptor co-receptor (Orco) of *Culex pipiens pallens* (CpOrco) were sequenced based on the comparison of conserved domains of *Culex quinquefasciatus* Orco (CqOrco). Genes encoding ribosomal protein (AaRps7 and DmRp49) were used as controls for RT-PCR experiments.

Gene name	Accession number*	Experiment purpose		Primer sequence	Product size (bp)
AaOrco	AAEL005776	Full coding sequence for calcium imaging	F	5'-ATGAACGTCCAACCGACAAAG-3'	1437
			R	5'-TTATTTCAACTGCACCAACACC-3'	
		Restriction	F	5'-TTAGGCGGCCGCTATGAACGTCCAACCGACAAAG-3'	1460
			R	5'-AAGCTCTAGATTATTTCAACTGCACCAACACC-3'	
AaOr8	AAEL012254	RT-PCR, qRT-PCR	F	5'-GCTCGGTAATAATCTGTCTGG-3'	144
			R	5'-TCGCTTCAATCAATTTGTTTCC-3'	
		RNA probes for *in situ* hybridization	F	5'-GTTCAACCTCAGCACGGATT-3'	551
			R	5'-AGATGGTCTTCAGCAAGGTG-3'	
		Full coding sequence for calcium imaging	F	5'-ATGGGAGGTAAGTTCTCAATAAAT-3'	1269
			R	5'-TCACTTCTGACTTGGTTCATAGAT-3'	
		Point mutation (AaOr8-S167A)	F	5'-TCGAGTCGCAAATGGTGATGAAGG-3'	1269
			R	5'-CCTTCATCACCATTTGCGACTCG-3'	
		Restriction	F	5'-TTAGGCGGCCGCTATGGGAGGTAAGTTCTCAATAAAT-3'	1292
			R	5'-AAGCTCTAGATCACTTCTGACTTGGTTCATAGAT-3'	
		dsRNA construction	F	5'-TAATACGACTCACTATAGGGGAACTTCACGAACTTTTTGG-3'	663
			R	5'-TAATACGACTCACTATAGGGTATTGGACGTAGCTTGGAA-3'	
AaOr49	AAEL001303	RT-PCR	F	5'-ATACTGTTCGCCGTCCTCAT-3'	146
			R	5'-AACTGGATCGCTTTCTGCAC-3'	
		qRT-PCR	F	5'-TGCCAACTCAATCTACAGC-3'	141
			R	5'-CCGAATCGTCCAATGTCTAC-3'	
		RNA probes for *in situ* hybridization	F	5'-AACAAGTTCTACGGGCTGGA-3'	560
			R	5'-AACTGGATCGCTTTCTGCAC-3'	
		Full coding sequence for calcium imaging	F	5'-ATGCTATTCGCGCGCTGTTTC-3'	1206
			R	5'-TTAGAATCGTTCCTTCAGTATCAAG-3'	

		Restriction	F	5'-TTAGGCGGCCGCTATGCTATTCGCGCGCTGTTTC-3'	1229
			R	5'-AAGCTCTAGATTAGAATCGTTCCTTCAGTATCAAG-3'	
		dsRNA construction	F	5'-TAATACGACTCACTATAGGGCGGAAGCTGTTTGATATTCT-3'	631
			R	5'-TAATACGACTCACTATAGGGCCAGGGATAGCTGTAGATTG-3'	
		Point mutation (AaOr49-Y190A)	F	5'-CACTACATAATCGCCATGATGATGCTGAC-3'	1206
			R	5'-GTCAGCATCATCATGGCGATTATGTAGTG-3'	
		Point mutation (AaOr49-V168M)	F	5'-CGATCGAGTTCATGCTGCCGCTGG-3'	1206
			R	5'-CCAGCGGCAGCATGAACTCGATCG-3'	
AaOr2	AAEL005999	RT-PCR	F	5'-CTTGCGCTACCAAAAGTCG-3'	145
			R	5'-AGATGGTCTTCAGCAAGGTG-3'	
AaOr3	AAEL017138	RT-PCR	F	5'-GCGTTCTGATGTGGTGTTTG-3'	191
			R	5'-AGGAGTCTCGTGCCAGGTAA-3'	
AaOr4	AAEL015147	RT-PCR	F	5'-TACCGCAGGCATTACTCTGA-3'	168
			R	5'-ATTCTGTTGGCTCACCTTCG-3'	
AaOr5	AAEL011843	RT-PCR	F	5'-GATGTGCATGCAGCTTTACC-3'	196
			R	5'-ATTCTGTTGGCTCACCTTCG-3'	
AaOr6	AAEL017548	RT-PCR	F	5'-TGCCACGATAGGATGAACAA-3'	200
			R	5'-CTGCTTTCGACAATGTGATG-3'	
AaOr7	AAEL005776	RT-PCR	F	5'-CCACTGGTATGACGGTTCC-3'	171
			R	5'-TTCAACTGCACCAACACCA-3'	
AaOr15	AAEL008448	RT-PCR	F	5'-TCAGATCATCGACTGCGTTC-3'	188
			R	5'-GGCAGTCAATAATGGCTCGT-3'	
AaOr46	AAEL017174	RT-PCR	F	5'-GAATGTGCGTTGTTACGGTCT-3'	185
			R	5'-GCGTTCCGTACATAGCATCTG-3'	
AaOr47	AAEL017079	RT-PCR	F	5'-CCGGATACGCACTAACCATT-3'	169
			R	5'-CACAGCATTGCAAACATTCC-3'	
AaOr55	AAEL010415	RT-PCR	F	5'-CATGCTACTTGCTGTTCGTCA-3'	185
			R	5'-AGGCATTCGCATGTACCAG-3'	
AaOr56	AAEL017534	RT-PCR	F	5'-GTTTTCGATGTTGGGAGCTG-3'	144
			R	5'-ATGCCAACTGGTCGATTTGT-3'	
AaRPS7	AAEL009496	RT-PCR	F	5'-CTGGAGGATCTGGTCTTC-3'	117
			R	5'-GTGTTCAATGGTGGTCTG-3'	
CpOrco	DQ231246.1** (Culex q. Orco)	RT-PCR	F	5'-GACTGGGGTGCCCAGTTC-3'	559
			R	5'-GCACCAACACCATGAAGTAGG-3'	
DmOrco	FBgn0037324*	RT-PCR	F	5'-GTCCTGGTTCCGGAAAGCCTC -3'	508

	**		R	5'-AGGGATGCGTGTTCACCTGG -3'	
DmRP49	AAB51389***	RT-PCR	F<br>R	5'-AGGGTATCGACAACAGAG-3'<br>5'-CACCAGGAACTTCTTGAATC-3'	122
AmOrco	GB19990#	RT-PCR	F<br>R	5'-TCACCATGCTCTTCTTCACG-3'<br>5'-TCGAAGATCCATGTCCAACA -3'	635
AaTRPV - nan-like	AAEL001123	RT-PCR	F<br>R	5'-TCGGGTTGAGATGTTTTTCC-3'<br>5'-TCGAAGATCCATGTCCAACA -3'	578
AaTRPV- iav like	AAEL009258	RT-PCR	F<br>R	5'-CGATTACGATTACGCCGACT-3'<br>5'-GTTTGGAGGGGTCGATTGT-3'	605
AaTRPA1	AAEL009419	RT-PCR	F<br>R	5'-TGCTTTCGGCTTAGCATTTT-3'<br>5'-CTGAACGATCAAACGAAGCA-3'	616

*Genome database for *Ae. aegypti:* https://www.vectorbase.org/organisms/aedes-aegypti.

**Genome database for *Culex quinquefasciatus*: https://www.vectorbase.org/organisms/culex-quinquefasciatus: Partial sequence of *Culex pipiens pallens* Orco (CpOrco), which was cloned by primer sets from the sequence of *Culex quinquefasciatus* Orco (CqOrco), was turned out to be perfectly matched each other (data not shown).

***Flybase database: http://flybase.org/

#Beebase: http://hymenopteragenome.org/beebase/
